# PBZGNet: A Novel Defect Detection Network for Substation Equipment Based on Gradual Parallel Branch Architecture

**DOI:** 10.3390/s26010300

**Published:** 2026-01-02

**Authors:** Mintao Hu, Yang Zhuang, Jiahao Wang, Yaoyi Hu, Desheng Sun, Dawei Xu, Yongjie Zhai

**Affiliations:** 1 College of Computer and Information Engineering (College of Artificial Intelligence), Nanjing Tech University, Nanjing 211816, China; 2 Department of Automation, North China Electric Power University, Baoding 071003, China; 3 Hangzhou International Innovation Institute, Beihang University, Hangzhou 311115, China

**Keywords:** substation equipment, feature fusion, attention mechanism, defect detection

## Abstract

As power systems expand and grow smarter, the safe and steady operation of substation equipment has become a prerequisite for grid reliability. In cluttered substation scenes, however, existing deep learning detectors still struggle with small targets, multi-scale feature fusion, and precise localization. To overcome these limitations, we introduce PBZGNet, a defect-detection network that couples a gradual parallel-branch backbone, a zoom-fusion neck, and a global channel-recalibration module. First, BiCoreNet is embedded in the feature extractor: dual-core parallel paths, reversible residual links, and channel recalibration cooperate to mine fault-sensitive cues. Second, cross-scale ZFusion and Concat-CBFuse are dynamically merged so that no scale loses information; a hierarchical composite feature pyramid is then formed, strengthening the representation of both complex objects and tiny flaws. Third, an attention-guided decoupled detection head (ADHead) refines responses to obscured and minute defect patterns. Finally, within the Generalized Focal Loss framework, a quality rating scheme suppresses background interference while distribution regression sharpens the localization of small targets. Across all scales, PBZGNet clearly outperforms YOLOv11. Its lightweight variant, PBZGNet-n, attains 83.9% mAP@50 with only 2.91 M parameters and 7.7 GFLOPs—9.3% above YOLOv11-n. The full PBZGNet surpasses the current best substation model, YOLO-SD, by 7.3% mAP@50, setting a new state of the art (SOTA).

## 1. Introduction

### 1.1. Background and Significance

Electrical substations form the backbone of modern power systems, stepping voltages up or down, steering power flows, and shielding the network from faults that stretch from generation to final consumption [1,2]. When substation equipment falters, the repercussions ripple outward, undermining the grid’s safety, stability, and overall efficiency [3]. As cities expand and industry intensifies, an uninterrupted substation service is no longer a convenience but a necessity for homes, hospitals, data centers, transport hubs, and emergency systems alike [4]. The rising share of renewables adds another layer of uncertainty, amplifying voltage swings and control tasks that make it harder to keep substations running smoothly [5,6]. Because of these pressures, power utilities worldwide are refocusing their efforts on earlier defect detection and smarter maintenance routines [7].

Conventional substation inspection—manual checks, infrared imaging, and partial-discharge tests—demands extensive engineering judgment, yielding slow, subjective surveys that frequently overlook faint flaws [8,9,10]. By coupling artificial intelligence with computer vision, recent work has automated fault detection, sharply raising both speed and accuracy [11,12]. Yet field deployment remains problematic. Corrosion, scratches, and minor structural damage present as diminutive features that general object detectors struggle to isolate [13], while shifting light, cluttered backgrounds, and varied equipment layouts further erode reliability [14].

Although YOLO and Faster R-CNN [15,16] have shown strong performance on generic object-detection benchmarks, their off-the-shelf versions cope poorly with the fine-grained appearance of electrical-equipment faults and demand too much computation for real-time inference on edge devices. In addition, in recent years, there have been technical means to achieve defect detection by combining edge intelligence and digital twins [17,18]. However, in order to achieve “zero-latency” real-time fault monitoring, we still need to design a deep learning detector that can enable the detection system to go off the cloud and process high-resolution visual data locally in real time.

### 1.2. Main Contributions

To overcome the shortcomings of current defect-detection techniques in cluttered substation scenes, we introduce PBZGNet. PBZGNet is a Substation Defect Detection Model integrating three core innovations: BiCoreNet, ZFusion-Neck, and ADHead. PBZGNet delivers reliable defect localization across six typical substation settings, recording 83.9% mAP@50 and 83.4% precision with merely 2.91 million parameters—superior to other state-of-the-art models of similar size. The principal contributions are outlined below:

(1) BiCoreNet for enhancing the characterization of micro defects: The clutter of the substation background covers up weak or small defects; in order to solve the problem of being unable to accurately capture small defects, we propose BiCoreNet. BiCoreNet uses the Gradual Parallel-Branch Architecture to enrich the semantic space clues layer by layer, and reweights the feature map through the Global Channel-Recalibration Module to make the defect-related channels more prominent and reduce the influence of irrelevant background and noise. By synergistically enhancing the information channel and suppressing noise, the network can identify multi-scale fine defects with different shapes without additional supervision.
(2) AvgDown for efficient feature downsampling: In order to reduce the amount of computation without damaging the quality of features, we propose an average-pooled downsampling layer, AvgDown, based on an attention guidance mechanism. Avgdown retains important feature information through an attention mechanism, thus inhibiting information loss. Compared with standard pooling or strided convolution, this method can reduce complexity by 25–30%, while preserving the fine structure of the defect region as completely as possible.
(3) ZFusion-Neck for multi-scale feature aggregation: Defect sizes in our dataset span a considerable range in size, from rust spots a few pixels across to large structural faults filling half the image. A single-resolution feature map cannot cover that range well. ZFusion-Neck rescales features up and down, redistributes channel capacity at each pyramid level, and merges them back. Shallow layers keep texture; deep layers add context. We found this neck particularly helpful for medium-sized defects that neither the high-resolution nor the low-resolution branch alone could localize cleanly.
(4) ADHead for robust detection in cluttered backgrounds: Overlapping structures, reflections, and uneven lighting all add noise to the feature maps that reach this stage. ADHead applies channel attention that looks at pairwise channel relationships, then rescales each channel by how strongly it correlates with known defect patterns from training. This makes the head less sensitive to background clutter and improves confidence scores for partially occluded defects.

The rest of the paper is organized as follows. Section 2 surveys the literature on object-detection algorithms, attention mechanisms, and defect detection in power equipment. Section 3 details the proposed PBZGNet approach, covering BiCoreNet, ZFusion-Neck, and ADHead. Section 4 outlines the experiments, benchmarks, and comparative results. Section 5 summarizes the findings and suggests future work. Lastly, Section 6 reviews the substation defect-detection model developed in this study.

## 2. Related Works

### 2.1. Defect Detection Methods in Power Equipment

Detecting defects in power equipment is essential for keeping substations reliable and safe. Manual inspections and conventional image-processing tools—edge detection, template matching, morphological operations—struggle with faint flaws, shifting light, and cluttered scenes [19,20]. Deep learning models, by contrast, have pushed accuracy and robustness well beyond these earlier limits.

YOLO-based object detectors have become the default choice for industrial defect inspection because they run in real time and train end-to-end. Shi et al. [21] presented SONet, a YOLOv8 variant tuned for small objects in power-line inspection, pushing mAP@50 6.7% above the baseline YOLOv8s. Lu et al. [16] adapted YOLOv5s to transmission-line faults and showed both higher precision and faster inference under challenging lighting. Gao et al. [22] embedded Transformer blocks into YOLOv10 to capture global context, reaching 84.2% mAP@50 at 78 FPS and outperforming conventional CNN versions. Chen et al. [23] equipped YOLOv9 with a multi-scale attention module, EMA, that fuses features across layers and yields 1.5× better precision-latency trade-off on substation data. Meng et al. [24] compressed and distilled a YOLO model for insulators, trimming parameters to 3.1 M while keeping accuracy intact. Luo et al. [25] refined YOLO for tiny bolt defects, boosting mAP@50 by 5.3% on targets smaller than 16 × 16 pixels.

Transformer-based architectures, valued for their capacity to model long-range dependencies and encode global context, have also delivered strong results in defect detection. Li et al. [26] combined Swin Transformer with CNN in a hybrid model that raised defect recognition accuracy by 7.4% mAP@50 over conventional CNNs on complex surfaces. Gao et al. [27] presented Cas-VSwin Transformer, which employs hierarchical self-attention to strengthen the representation of subtle defects and reached 85.6% mAP@50 on industrial datasets. Wang et al. [28] introduced the Pyramid Vision Transformer (PVT), setting a new benchmark for small-scale defect detection by surpassing ResNet-FPN baselines by 8.2% mAP@50. Transformer models adapted for power-quality disturbance detection [29] run faster than 60 FPS, confirming their suitability for time-critical monitoring systems.

Beyond YOLO and Transformer models, CNN-based and hybrid designs continue to advance defect detection in power equipment. Single Shot MultiBox Detector (SSD) networks, for example, are valued for completing inference in one forward pass. Zhao et al. [30] introduced ShuffleNet-CA-SSD, a compact SSD variant that embeds channel attention modules to spot defects on wind-turbine blades; the light footprint makes it practical for IoT and edge devices. Coupling SSD with strong augmentation—optical-flow-based methods, in particular—has also improved defect localization when operating conditions are harsh.

Cheng et al. [31] refined R-CNN variants, lifting detection accuracy for assorted power-equipment parts. Luo et al. [25] tailored a CNN to spot ultra-small bolt flaws on transmission lines. Chen et al. [23] deployed ShuffleNet coupled with channel attention, enabling fast yet accurate defect tagging in electrical devices. Lv et al. [32] embedded attention modules inside DenseNet, steadying performance under shifting outdoor conditions.

### 2.2. Performance Enhancement Strategies for Object Detection Methods

Two-stage detectors—especially the R-CNN family—have pushed accuracy steadily upward. Tang et al. [33] upgraded the basic architecture with a feature pyramid network, allowing for multi-scale flaws to be captured in a single pass. Zhang et al. [34] paired RetinaNet with a sequence-transduction module, lifting accuracy when imaging conditions vary. Zhang et al. [35] benchmarked Mask R-CNN on wind-turbine blade inspection, proposed new evaluation metrics, and reported high precision for defect classification. Although these two-stage approaches deliver strong accuracy, their computational load often prevents real-time deployment.

Attention modules help detection networks zoom in on the defect cues that matter. Meng et al. [24] built SlimNeckNet, a channel-attention block that lifts accuracy without adding heavy computation. Zhang and Qing et al. [36] introduced Shuffle Attention (SA-Net), blending spatial and channel cues so that defects are pinned down more precisely. Lv et al. [32] and Li et al. [37] pushed performance further with hybrid attention, especially when the background is clustered.

Recent network designs—Swin Transformer [26], Pyramid Vision Transformer (PVT) [28], Path Aggregation Network (PANet) [38], and Bidirectional Feature Pyramid Network (BiFPN) [39]—have greatly improved multi-scale fusion, making faint, small defects easier to spot. Lightweight backbones such as GhostNet [40] and MobileNetV3 [41] couple low latency with solid accuracy, so they fit edge devices that must run in real time.

### 2.3. Summary and Research Gaps

Although defect detection has advanced, subtle flaws still slip through when the background is clustered, and the scale varies widely. Edge hardware further limits what can run in real time. To address these limitations, this research introduces PBZGNet, a dual-backbone network with spatial attention and a new fusion path, designed to keep substation inspections both accurate and light enough for field devices.

## 3. Materials and Methods

### 3.1. Data Acquisition and Processing

In this study, a private dataset covering a wide range of operating conditions was constructed by collecting substation defect images across various time periods and geographic locations. To ensure the scientific rigor of the experiments, the dataset was strictly partitioned into training, validation, and testing sets. The model underwent parameter optimization and hyperparameter tuning on the training and validation sets, while final performance evaluations were conducted on the testing set. Furthermore, to evaluate the generalization ability and robustness of PBZGNet beyond the private dataset, an independent public dataset was introduced as a cross-domain evaluation benchmark. This additional step aims to verify the model’s detection efficacy across diverse equipment layouts and varying ambient lighting conditions.

#### 3.1.1. Private Dataset Construction

**Figure 1:**
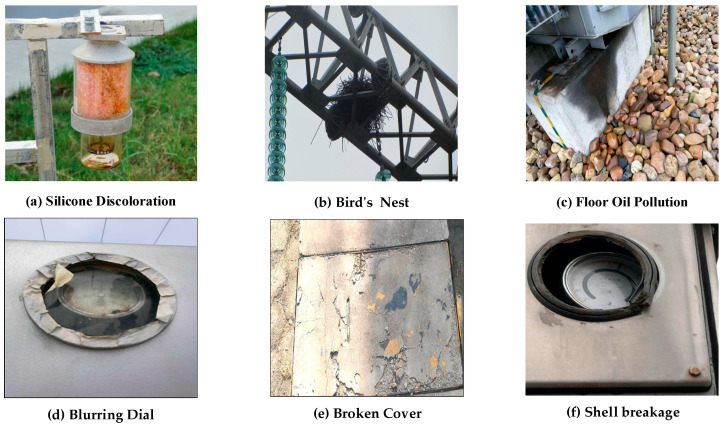
DatasetSample images of substation faults in a specific dataset.

Figure 1 shows sample images of substation defects examined in this study. Data were collected by installing image-acquisition systems at 220 kV and 500 kV substations in Henan, Shandong, and Jiangsu provinces, China. Each outdoor switchyard covers roughly 1000–1500 m^2^ of cement and gravel and contains bus bridges, surge arresters, transformer tanks, and other equipment.

To capture defect features from multiple viewpoints, three acquisition devices were deployed:

(1) Fixed high-definition cameras were installed at key locations—main transformers, circuit breakers, and busway corridors—3 m above ground and oriented almost perpendicular to the equipment surface; they recorded one image every five minutes.
(2) DJI Mavic 3 Pro drones carried 4K cameras and hovered between 10 m and 30 m. A 5 m × 5 m grid guided the flight lines, keeping both along-track and across-track overlap above 60%.
(3) Inspectors mounted a GoPro Hero 11 Black 1.2–1.5 m above the floor and recorded from 1 to 3 m away to keep every detail sharp.

Data were gathered from 1 June 2022 to 31 October 2024, covering sunny, cloudy, and post-rain scenes and the changing light of four seasons. To limit glare and deep shadows, fixed-camera and hand-held shots were scheduled mainly between 09:00 and 11:30 and 13:30 and 16:30 each day. Drone flights were flown only when wind speed stayed at or below 5 m s^−1^ and visibility exceeded 2 km.

Of the 8680 raw images—resolutions ranging from 1920 × 1080 to 4096 × 2160—those that were blurred, motion-streaked, or out of focus were removed, as were views blocked by pipes, debris, or pedestrians. Frames that clearly showed defects, such as cracks or corrosion, were kept. The screening yielded 6131 clean, valid photographs, providing a solid base for training the substation-equipment defect-recognition model (as Figure 1).

As shown in Table 1, among the 6131 valid images, defect types included: Bird’s Nest (1094 photographs), Blurred Dial (1143 images), Floor Oil Pollution (769 images), Shell Breakage (658 images), Broken Cover (1039 images), and Silicone Discoloration (1428 images).

The flaw size distribution displayed clear multi-scale characteristics:

(1) Minor faults (<32 × 32 pixels): 45.3% percentage, average dimension 24 × 28 pixels.
(2) Medium faults (32 × 32 to 96 × 96 pixels): 38.7% percentage, average size 58 × 64 pixels.
(3) Large target flaws (>96 × 96 pixels): 16.0% proportion, average dimensions 156 × 142 pixels.

Moreover, fault images in the substation apparatus displayed the subsequent characteristics:

(1) Complicated backgrounds: Substation settings comprised huge metallic structures, intertwined lines, and dense apparatus, leading to significant background interference.
(2) Notable lighting variations: These comprised direct intense illumination, shadow obstruction, and nocturnal infrared conditions.
(3) Varied fault morphologies: Comparable faults displayed unique visual attributes across various apparatus and phases of development.
(4) Elevated ratio of minor objectives: Almost fifty percent of flaws were smaller than 32 × 32 pixels, presenting considerable identification difficulties.
(5) Class imbalance: Certain critical problems (e.g., casing damage) exhibited a lower frequency of samples.

The dataset was split into training (3678 images), validation (1226 images), and test (1227 images) subsets at a 6:2:2 ratio, and the proportion of each defect class was held constant across the splits to limit evaluation bias caused by class imbalance.

#### 3.1.2. Public Datasets

**Figure 2:**
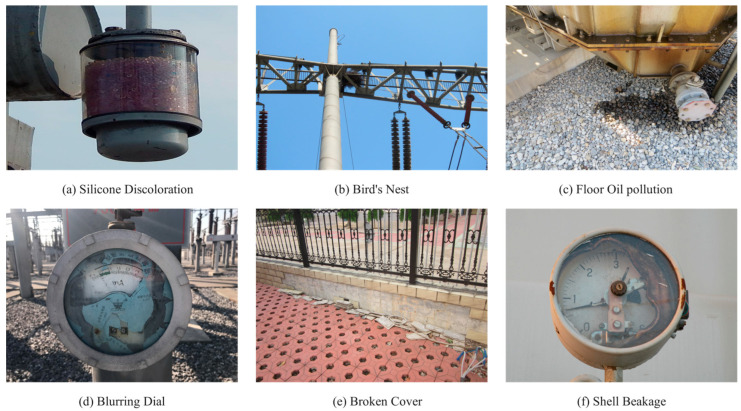
DatasetSample images of substation faults in a public dataset.

As shown in Table 2, this study drew on 1308 photographs from the publicly available collection [42]. Of the 1308 valid photographs, the distribution was as follows: Silicone Discoloration (291), Bird’s Nest (220), Floor Oil Pollution (262), Blurred Dial (273), Broken Cover (184), and Shell Breakage (78). The public set was used solely to test the generalization of the proposed model and therefore served only as the validation split. Figure 2 shows sample images from this dataset [42].

### 3.2. PBZGNet for Substation Defect Detection

When deep networks are deployed for substation defect inspection, they must cope with cluttered scenes, objects of widely different appearance, and the tight latency demanded by live monitoring. Shrinking the model only amplifies the dilemma: every parameter trimmed risks eroding accuracy. Single-backbone architectures compound the problem, because each pooling step discards detail, weakening the gradients that steer training and dragging both convergence and final precision downward.

**Figure 3:**
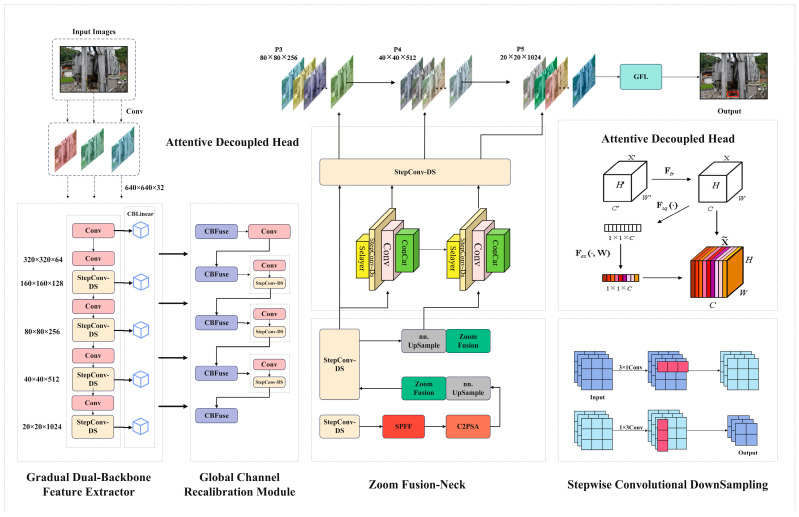
PBZGNet architecture diagram.

To tackle the difficulty of spotting substation defects against cluttered backgrounds, we put forward an object-detection model built on YOLOv11. The network, named PBZGNet, improves YOLOv11 through a gradual parallel-branch structure (ZFusion-Neck), a global channel recalibration module, and an ADHead detector. By inserting auxiliary reversible branches and a programmable gradient-information path, PBZGNet curbs information loss in deep layers without raising inference cost. Figure 3 shows that PBZGNet contains three components designed for defect inspection in complex substation scenes: BiCoreNet, ZFusion-Neck, and ADHead. BiCoreNet itself combines the GPBA and GCRM blocks.

Figure 3 shows that PBZGNet locates the six substation defects defined in Section 3.1 through a three-layer cooperative pipeline. GPBA first stacks CSP—a residual block refined from YOLOv11—and StepConv-DS, an asymmetric downsampling unit. The image is progressively shrunk to four scales (160 × 160 × 128, 80 × 80 × 256, 40 × 40 × 512, 20 × 20 × 1024), cutting computation yet preserving a receptive field close to that of a 3 × 3 convolution. At the end of every residual path, a linear layer widens the channel count by 25%, enriching the semantic space. Next, the GCRM module pools each dimension globally, captures long-range context and subtle cues, and feeds the result to a two-layer auto-gating network that reweighs the channels. The recalibrated features are then merged back with the original maps, letting the network highlight multi-scale, multi-shaped fault patterns in substation imagery.

ZFusion-Neck first employs an AvgDown operation with stride = 2 to align the four feature maps (160 × 160 × 128, 80 × 80 × 256, 40 × 40 × 512, 20 × 20 × 1024) delivered by the gradient feature extraction network to a uniform resolution, thereby reducing scale discrepancies in subsequent fusion stages.

Then, we perform the ZFusion operation on each pair of adjacent scales: take the high-resolution image as the benchmark, upsample the low-resolution image to the appropriate size, and downsample the reference image accordingly. The two are spliced along the channel dimension to retain the shallow texture and deep semantic information at the same time. After each fusion, we added AvgDown, BatchNorm, and ReLU layers to eliminate redundant information, balance channel response, and reduce the computational load. Finally, after feature fusion, we let the features be processed by BatchNorm and ReLU again to form a composite feature pyramid with perfect structure. The composite feature pyramid effectively enhances the ability of the model to express microdefects and complex contours.

ADHead uses the dynamic channel reallocation unit to receive the multi-scale fusion results of the composite feature pyramid. The module can allocate appropriate channel weights for defects of different sizes and shapes through autonomous learning to ensure that both fine defects (such as microcracks, local corrosion) and large defects (such as large-area fracture) can be optimally handled. The regression branch adopts deformable convolution, which can dynamically adjust the shape of the convolution kernel in real time by learning the offset field, and accurately fit the geometric shape of irregular defects. The classification and location branches embed a channel attention mechanism and a spatial attention mechanism in the decoupled lightweight network, respectively; the former enhances category discrimination through adaptive focusing channel difference, while the latter emphasizes spatial positions to refine center and bounding box coordinates. All scales simultaneously output category confidence scores and coordinate offsets, with redundant boxes filtered via Soft-NMS. This enables simultaneous detection of six substation defect categories under complex electrical environments and variable lighting conditions. Subsequent sections detail the architecture and enhancements module by module.

### 3.3. Implementation Methods

#### 3.3.1. BiCoreNet

To improve the model’s ability to represent defects that vary in scale and shape, we introduce BiCoreNet, a dual-branch feature extractor shown in Figure 3. Its blueprint rests on three ideas: dual-core parallel processing, reversible residual connections, and channel recalibration. The main and companion branches work together, pulling clean signals for micro-cracks, pitting, large fractures, and delamination from the image. Borrowing from information-bottleneck and reversible-network studies, the design avoids the usual loss of gradients and detail that accompanies deep stacking: reversible links let error flow unaltered from the deepest layer back to the shallowest, while the auxiliary path continuously enriches the primary one. This dual route lets the network strengthen and separate multi-scale patterns early, so it can spot both tiny fissures and full insulator breaks in one pass.

Deepening the network risks gradient decay, we add the CBLinear module. It parcels features between trunk and side branches at several channel scales, as shown in Algorithm 1. A stack of grouped 1 × 1 convolutions first splits the deep trunk output into feature maps whose channel counts differ; the maps are then fed to the corresponding auxiliary layers in the original order. The scheme keeps coarse-to-fine information intact and opens multiple gradient highways, markedly slowing vanishing signals.
**Algorithm 1** CBLinear Forward ProcessDef CBLinearRequire: Input feature X∈ℝ^{B × C_in × H × W}; Total number of convolution kernel channels G; Group size list [g_1,…,g_n] Ensure: Output grouped features{F_i∈ℝ^{B × g_i × H × W}}1.Initialize 1 × 1 convolution parameters W∈ℝ^{G × C_in × 1 × 1} 2.Store batching options according to [g_1,…,g_n] 3.X’ ← Conv1 × 1(X; W) 4.{F_1,…,F_n} ← Split(X’, [g_1,…,g_n], dim = 1 5.Return ordered group {F_i}

Designate the output of the $k_{th}$ layer of the main tree be $X\in R^{C_{in}\times H\times W}$, and the output $F_{i}\in R^{g_{i}\times H\times W}$ corresponding to the $i_{th}$ group in CBLinear can be expressed as:

(1) $$F_{i}\;=(W_{i}*X{)}_{\;C_{i-1}:C_{i}},\;\;C_{i}=\sum_{j=1}^{i}g_{j},$$

where $W_{i}\in R^{g_{i}\times C_{\mathrm{in}}\times 1\times 1}\;$ represents the convolution sub-kernel for the $i_{th}$group, ∗ denotes per-channel convolution. The slicing operation $({W_{i}*X)}_{C_{i-1}:C_{i}}$ extracts the corresponding channel interval from the overall mapping output.

During backpropagation, CBLinear distributes the loss gradient $\nabla_{X}L$ across all $F_{i}$ according to the same linear mapping:

(2) $$\nabla_{F_{i}}L\;=\;W_{i}^{⊤}\;\nabla_{X}L\;,$$

forming *n* parallel gradient backpropagation paths. Experiments demonstrate that this strategy increases the average gradient norm in the front-end convolutional layers rises roughly threefold, markedly curbing vanishing gradients in deep architectures.

The first stage of BiCoreNet employs GPBA, with the core idea being to run two lightweight residual backbones in parallel, thereby simultaneously capturing global semantics and local texture. Each backbone alternately stacks CSP residual blocks (Cross-Stage Partial Residual Block) with StepConv-DS (Stepwise Convolutional DownSampling). The CSP module first splits the input features into two streams: one feeds directly into CBLinear for feature extraction, while the other undergoes residual convolution before being concatenated back along the channel dimension. This method reduces redundant computation while preserving original information. StepConv-DS realizes downsampling through asymmetric convolution (1 × 3 followed by 3 × 1 convolution with a step size of 2), which significantly reduces the number of parameters compared with the direct use of a 3 × 3 convolution kernel.

Designate the input feature map as $X\in R^{C_{\mathrm{in}}\times H\times W}$, output $Y\in R^{C_{\mathrm{out}}\times \frac{H}{2}\times \frac{W}{2}}$,

(3) $$U={\mathrm{Conv}}_{1\times 3,\;\mathrm{stride}=1}\left(X;\;W^{\left(1\right)}\right),\;$$

(4) $$\;Y=\mathrm{ReLU}\left(\mathrm{BatchNorm}\left({\mathrm{Conv}}_{3\times 1,\;\mathrm{stride}=2}\left(U;\;W^{\left(2\right)}\right)\right)\right),$$

$InStepConv-DS{,\;\mathrm{Conv}}_{1\times 3,\;\mathrm{stride}=1}(X;\;W^{\left(1\right)})$, a 1 × 3 convolution operation with channel size matching, is adopted. ${\mathrm{Conv}}_{3\times 1,\;\mathrm{stride}=2}(U;\;W^{\left(2\right)})$ reduces the spatial resolution by half through 3 × 1 convolution with Step = 2; subsequently, the feature map is normalized with BatchNorm and activated by ReLU.

**Figure 4:**
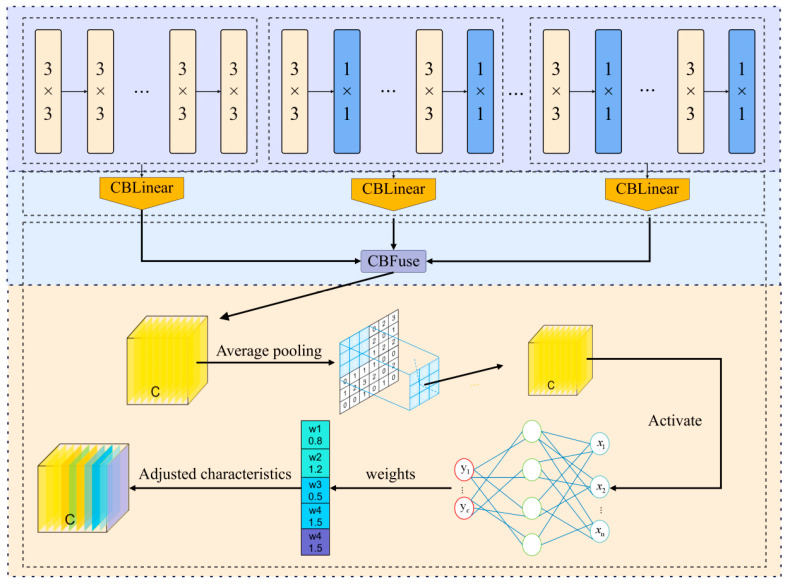
Global channel recalibration module.

In the second stage, the global channel weight recalibration mechanism (GCRM, as shown in Figure 4) is introduced for the global recalibration of channel weights after fusion. After multi-source feature fusion, the correlation of each channel to defect detection is different, so it is necessary to strengthen the effective response and suppress noise through adaptive weight adjustment. GCRM uses the principle of an SE attention mechanism for reference and uses global statistics to re-score the channel. The fused input feature graph is defined as $F\in R^{C\times H\times W}$. Initially, apply AvgDown to $z\in R^{C}$ to derive the channel description vector, wherein the $c_{\mathrm{th}}$ component corresponds to $z_{c}=\frac{1}{H\times W}\sum_{i=1}^{H}\sum_{j=1}^{W}F_{c}\left(i,j\right)$. Subsequently, a two-layer fully linked bottleneck network executes a nonlinear transformation on z, producing the channel weights $s=\sigma (W_{2},\delta (W_{1},z))$, where δ(⋅) represents the ReLU activation function and σ(⋅) denotes the Sigmoid function. Ultimately, s is transmitted back to the initial feature map, recalibrating each channel: $\tilde{F}c=s_{c}\cdot \mathrm{Fc}$. This phase amplifies globally discriminative feature channels while diminishing irrelevant or redundant ones. The GCRM design guarantees the global integration of information from fused features prior to advancing to further phases. This enhances the signal-to-noise ratio of feature representation, allowing for the subsequent detection heads to concentrate more effectively on defect-related salient features. As a result, the accuracy and reliability of detection are improved.

#### 3.3.2. Dynamic Fusion Mechanism Based on Concat–CBFuse and Cross-Scale ZFusion

Figure 4 illustrates that, in the feature fusion stage, PBZGNet first concatenates the primary feature along the channel axis and applies CBFuse to re-weight the resulting tensor together with N auxiliary features $X^{\left(m\right)}$,$\{X_{i}^{\left(a\right)}{\text{\}}}_{i=1}^{N}$. It then injects spatial attention at higher layers, after which ZFusion aligns and merges the multi-scale cues. The pipeline unfolds in three stages:

Stage 1: Channel Concatenation

All incoming feature maps are simply stacked along the channel dimension.

(5) $$F_{\mathrm{cat}}=[X^{\left(m\right)}\;\|\;X_{1}^{\left(a\right)}\;\|\;\cdots \;\|\;X_{N}^{\left(a\right)}]\;,$$

$\mathrm{where}\;C_{\mathrm{tot}}=C_{m}+\sum_{i=1}^{N}C_{a,i}$, and $F_{\mathrm{cat}}\in R^{C_{\mathrm{tot}}\times H\times W}$.

Step 2: The formula for dynamically regenerating CBFuse permissions is as follows:

$$F_{\mathrm{grp}}={\mathrm{GroupConv}}_{1\times 1}\left(F_{\mathrm{cat}};\;\mathrm{groups}=N+1\right)\;,\;$$

(6) $$\;z=\mathrm{GAP}\left(F_{\mathrm{grp}}\right)\in R^{C_{\mathrm{tot}}}\;,$$

$$s=\sigma (W_{2}\;\delta (W_{1}\;z)),\;\hat{F}=s⊙F_{\mathrm{grp}}\;,\;$$

where $W_{1}\in R^{\frac{C_{\mathrm{tot}}}{r}\times C_{\mathrm{tot}}},W_{2}\in R^{C_{\mathrm{tot}}\times \frac{C_{\mathrm{tot}}}{r}},r=16,\delta \;\mathrm{denotes}\;\mathrm{ReLU},\;\sigma \;\mathrm{denotes}\;\mathrm{Sigmoid}.$

In practical implementation, the forward process of CBFuse is as shown in Algorithm 2.
**Algorithm **2 CBFuse Forward PropagationDef CBLinearRequire: Input {X^(m), X^(a)_1,…,X^(a)_N} Ensure: Output Weighted features of each channel {X′_i}1.F_cat ← concat({X}, dim = 1) 2.conv ← Conv1 × 1(in = C_tot, out = C_tot, groups = N + 1) 3.F_grp ← conv(F_cat) 4.{X′_i} ← split(F_grp, [C_m,C_{a,1},…,C_{a,N}], dim = 1) 5.return {X′_i}

Step 3: Spatial Attention

Compute the spatial attention map $M_{sp}$ on the high-level fusion output $F^{\hat{F}}$:

$$M_{\mathrm{sp}}=\sigma (f^{1\times 1}\left(\left[\mathrm{AvgPool}\left(\hat{F}\right)\left\|\mathrm{MaxPool}\left(\hat{F}\right)\right]\right)\right),$$

(7) $$\tilde{F}=M_{\mathrm{sp}}⊙\hat{F}\;,$$

where $f^{1\times 1}\;\mathrm{denotes}\;1\times 1\;$ convolution operation.

#### 3.3.3. ZFusion-Neck

ZFusion-Neck was designed to collect multi-scale features and forward them to the detection head. Because substation defects vary in size and appearance, the network must merge cues from different levels—fine details from high-resolution layers and semantic context from low-resolution ones—at the same time. Classic FPNs usually add the layers element-wise, yet simple addition can erase or weaken signals at certain scales. To avoid this, ZFusion-Neck rescales each map (up or down) and then concatenates them, keeping the full spectrum of information. Starting from the three maps P3, P4, and P5, delivered by the earlier stages, we bring them to a common resolution (P4’s scale). Specifically, P5 is upsampled to match P4’s resolution, while P3 is downsampled to the same scale. These rescaled features are then concatenated channel-wise, preserving the full spectrum of multi-scale information in a single unified representation.

(8) $$\;P_{Z}=\Phi (\mathrm{Concat}\left(\mathrm{Up}\left(P_{5}\right),P_{4},\mathrm{Down}\left(P_{3}\right)\right)\;,$$

where

Up(·):Denotes bilinear interpolation or deconvolution upsampling;
$\mathrm{Down}\left(\cdot \right):\mathrm{Denotes}\;\mathrm{subsampling}\;\mathrm{with}\;\mathrm{average}\;\mathrm{pooling}\;\mathrm{characteristics};$
$\Phi \left(\cdot \right):\;\mathrm{Represents}\;\mathrm{an}\;\mathrm{adaptive}\;\mathrm{fusion}\;\mathrm{function}\;\mathrm{composed}\;\mathrm{of}\;1\times 1\;\mathrm{and}\;3\times 3\;\mathrm{convolutions}.$

The merged features are then further transformed and compressed by successive convolutional layers, integrating cues from diverse sources. ZFusion-Neck preserves raw information at multiple scales by concatenating mid- and high-level fusion outputs. Later convolutions learn to weigh multi-scale properties on the fly, enabling $F_{\mathrm{out}}$ to encompass multi-level spatial and semantic information.

Mid-level fusion:

$$P_{Z1}=\mathrm{Concat}(\mathrm{Up}(P_{5}),P_{4}),$$

(9) $$P_{Z1}^{\text{′}}=\sigma \left(W_{1}*P_{Z1}+b_{1}\right),$$

where * denotes convolution and σ represents the activation function (ReLU).

High-level fusion:

$$P_{Z2}=\mathrm{Concat}(\mathrm{Up}(P_{Z1}\text{′}),P_{3}),\;$$

(10) $$P_{\mathrm{out}}=\sigma \left(W_{2}*P_{Z2}+b_{2}\right).$$

We introduce cross-branch linear mapping and a feature concatenation layer (CBFuse) to let the parallel trunks exchange information. The mapping first rescales features of different resolutions into a common space, supplying multidimensional inputs for the subsequent concatenation. CBFuse then merges these aligned auxiliary signals with the main-stream features, preserving richer information and mitigating gradient vanishing.

Linear Mapping Layer:

(11) $${\tilde{P}}_{a}=W_{L}\cdot P_{a}+b_{L}\;.$$

Maps ancillary branch characteristics $P_{a}$ is in the same dimensional space as trunk characteristics $P_{m}$

Concat Layer:

(12) $$\;P_{\mathrm{CBFuse}}=\mathrm{Concat}\left({\tilde{P}}_{a},F_{m}\right),$$

and through convolutional layers, extract cross-branch semantic information to achieve bidirectional feature interaction.

#### 3.3.4. Attentive Decoupled Head

Substation defect detection hinges on spotting minute, localized cues—cracks, corrosion—that are easily buried in cluttered backgrounds or hidden behind other hardware. The head network, therefore, has to discriminate these faint signatures while actively downplaying surrounding noise.

**Figure 5:**
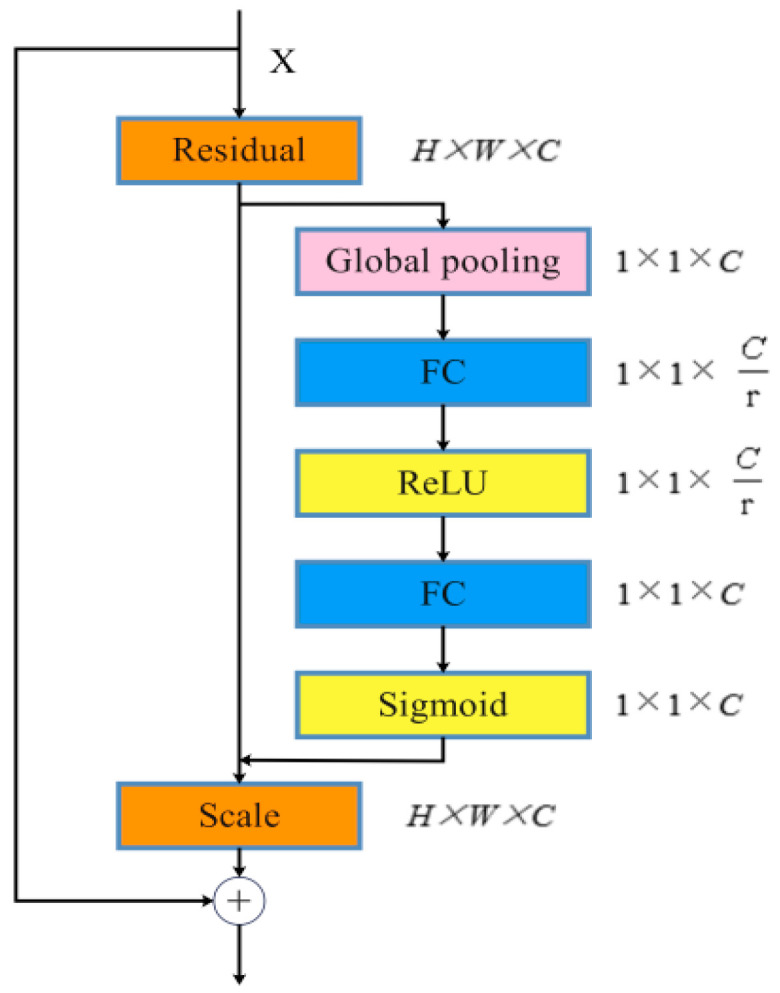
Defect-focused attention layer module.

Figure 5 shows the ADHead. By integrating the decoupling prediction with the lightweight channel attention block, the module enhances the sensitivity of the model to subtle defects without reducing the reasoning speed.

Adhead consists of three elements:

(1) Task Decoupling Framework (Decoupled Prediction Branches)

ADHead splits the detection head into two independent branches: the classification branch and the regression branch. The classification branch extracts defect type features while suppressing background interference, while the regression branch focuses on predicting the position and shape of the bounding box. By decoupling these two tasks, the design significantly reduces the gradient conflict between classification and positioning, so as to stabilize the training process and improve the recall rate of small targets.

(2) Channel Attention Module

ADHead enhances the ability of feature representation by capturing the dependencies between channels. ADhead will reassign appropriate weights to each channel in real time, so that the network can highlight the most informative channel while suppressing redundant information, so as to enhance its sensitivity to defect characteristics.

First, ADHead applies global average pooling to the input characteristic graph, averaging all spatial positions in each channel to obtain channel-level statistics.

(13) $$z_{c}=\mathrm{GAP}\left(F_{c}\right)=\frac{1}{H\times W}{\sum_{i=1}^{H}\sum_{j=1}^{W}F}_{c}\left(I,j\right)\;,$$

$F_{c}\left(i,j\right)$ represents the feature value position (i, j) in channel c, where H and W give the height and width of the characteristic graph, respectively; $z_{c}$ represents the global average pooling result of channel c.

After obtaining these channel characteristics, ADhead inputs them into the two-layer fully connected network to learn the nonlinear dependencies between channels more effectively. Finally, a weight set is output to adjust the weight of each channel.

(3) Feature Recalibration and Prediction

ADHead finally recalibrates the original feature map with the learned channel weights s, scaling each channel by its own factor. Informative channels are amplified while redundant ones are toned down, sharpening the detection of power defects in cluttered scenes and steering the model toward the key signatures that matter.

(14) $$F_{\mathrm{out}}=\sigma \left(W_{2}\cdot \mathrm{ReLU}\left(W_{1}\cdot z\right)\right)⊙F\;,$$

where W_1_ and W_2_ are the weight matrices of the two fully connected layers, respectively. ReLU(⋅)  introduces nonlinearity. σ(⋅) denotes the Sigmoid function, which squeezes the generated weights to the range [0, 1]. F is the input feature map, s is the channel-wise weight produced by the two fully connected layers, ⊙ denotes channel-wise multiplication, and F̃ is the recalibrated feature map.

Since substation defects are often hidden in the background of strong interference in the form of small defects, ADHead solves this problem by re-correcting the channel weight in real time. It enables the detector to capture the signal of small defects and suppress irrelevant activation. This real-time weight adjustment mechanism can also reduce the impact of light fluctuations and local occlusion, reduce the possibility of missed detection and false positives, and achieve more stable real-time identification.

#### 3.3.5. AvgDown Lightweight Downsampling Design

Traditional downsampling usually uses convolution with a step size of 2. This will cause every other pixel to be discarded in the process of downsampling. In the image of power equipment, this kind of processing is fatal, because it will erode the small defect features (hairline cracks, needle tip corrosion, structural defects that are difficult to distinguish by the naked eye) and affect the result of defect detection.

To reduce this loss, we propose the AvgDown module, which aggregates the neighborhood values before reducing the resolution through attention-directed average pooling instead of step convolution, so as to reduce the loss of detail.

Step 1: Spatial Information Aggregation

The method of using average pooling to aggregate the input characteristic graph is different from the traditional step convolution. This method can preserve the spatial distribution characteristics of input information more evenly and minimize the loss of information.

(15) $$F_{\mathrm{agg}}=\mathrm{AveragePooling}\left(X,\mathrm{factor}=2\right)\;,$$

where X is the input feature map, and $F_{\mathrm{agg}}$ is the aggregated feature map.

Step 2: Feature Transformation

The 1 × 1 convolution operation is used for the aggregation feature graph to adjust the number of channels and compress the dimension. This step not only reduces the computational load but also retains the key channel-level information and effectively suppresses redundant data.

(16) $$\;F_{\mathrm{trans}}=W_{1\times 1}\cdot F_{\mathrm{agg}}\;,$$

where $W_{1\times 1}$ is the 1 × 1 convolution kernel, and $F_{trans}$ is the transformed feature map.

Step 3: Nonlinear Activation

The transformed feature map is activated nonlinearly by the activation function of SiLU (Sigmoid-weighted linear unit) to enhance the expression ability of the network.

(17) $$F_{\mathrm{final}}=\mathrm{SiLU}\left(F_{\mathrm{trans}}\right)\;.$$

The AvgDown module enhances the characterization ability of the feature map by retaining more key details in the downsampling process. This ability is very important for detecting tiny substation defects such as cracks.

### 3.4. Loss Function Design

#### 3.4.1. Generalized Focal Loss

The defect identification of a substation requires that the target detector can locate defects with high accuracy, such as cracks, corrosion, etc. However, these defects differ greatly in size and shape. Although the standard intersection union IoU (such as CIoU or SIoU) can accurately regress the geometric shape, it ignores how the aspect ratio or scale of the target itself interferes with the regression process. Therefore, the results are often unsatisfactory.

In order to make up for this defect, we designed the Generalized Focus Loss (GFL) function, which combines the Quality Focus Loss (QFL) and the Distribution Focus Loss (DFL), so that the GFL function can strengthen the boundary box regression accuracy, significantly improve the multi-scale positioning ability and the positioning accuracy of parts with significant shape differences when facing the common complex targets in power equipment detection.

#### 3.4.2. Principles of QFL and DFL Functions

Because GFL embeds the object quality estimation into the standard focus loss and combines it with multi-task regression, it can guide the network to focus on the samples that are difficult to detect, so it enhances the sensitivity of the model to small objects and tiny defects.

(1) Quality Focal Loss (QFL)

Traditional loss functions often allow for the surplus of negative samples—background regions in particular—to dominate training, skewing the model. QFL counters this by assigning each positive anchor a quality score that reflects how tightly its predicted box matches the ground truth. Loss weights are then scaled by this score, steering the network toward the most reliable positives.

(18) $$L_{\mathrm{QFL}}=-\frac{1}{N}\sum_{i}q_{i}\left(1-p_{i}{)}^{\gamma }\log(p_{i}\right)\;,$$

where $q_{i}$ denotes the quality score of the positive sample, indicating how confident the model is in the target box; it is usually derived from the overlap between the predicted and ground-truth boxes, such as IoU. $p_{i}$ is the expected probability of the target class.γ is the focal-loss parameter that increases the weight of hard-to-classify examples.

(2) Distribution Focal Loss (DFL)

In object-bounding box regression tasks, conventional losses typically focus solely on the offset between the center point and the box dimensions. DFL incorporates additional positional distribution information through distribution modeling, embedding distributional knowledge into the regression process to enhance localisation accuracy. This approach represents bounding boxes as probability distributions, enabling the network to capture finer spatial cues.

The mathematical formulation of DFL is as follows:

(19) $$L_{\mathrm{DFL}}=-\frac{1}{N}\sum_{i}p_{i}\log\left(\frac{q_{i}}{\sum_{j}\exp(q_{j})}\right)\;,$$

where $q_{i}$ represents the distribution parameter of the target box, reflecting its spatial diffusion degree; $p_{i}$ represents the box regression probability, which is the probability that the predicted box locks onto the true box.

QFL guides the network to focus on high-confidence targets by assigning low-quality scores to background samples, thereby reducing the impact of background samples on training. DFL utilizes distribution-aware regression technology to improve the regression accuracy for small and geometrically complex objects. After integrating QFL and DFL, the GFL function can dynamically adjust weights in real-time, accurately capturing crack details while fully preserving large-scale damage features such as transformer oil tank deformation, thereby enhancing the multi-scale detection capability of substations.

## 4. Results

### 4.1. Model Training Parameters and Evaluation Metrics

This section provides an overview of the experimental setup, including comparative experiments, ablation experiments, and tests to verify generalization ability. All experiments were run on an Ubuntu 20.04.1 system equipped with an NVIDIA RTX A6000 GPU.

The specific training configurations are as follows:

(1) Input and Data Processing: All images were resized to a uniform resolution of 640×640 pixels for training and inference. Data augmentation strategies, including Mosaic, Mixup, random scaling, horizontal flipping, and HSV color space transformations, were applied to enhance the model’s robustness against varying lighting and cluttered backgrounds.
(2) Optimizer and Scheduler: We employed the Stochastic Gradient Descent (SGD) optimizer with a momentum of 0.937 and a weight decay of 0.0005. The learning rate (LR) followed a Cosine Annealing schedule with an initial value of 0.01.
(3) Warmup and EMA: A 3-epoch warmup phase was utilized at the start of training to stabilize the initial gradients of the BiCoreNet pathways. Exponential Moving Average (EMA) was enabled with a decay rate of 0.9999 to ensure weight stability and improve generalization.
(4) Loss and Post-processing: The Generalized Focal Loss (GFL) function was adopted, integrating QFL and DFL functions to optimize both classification quality and bounding box distribution. For inference, Soft-NMS was used for redundant box suppression with a confidence threshold of 0.25 and an IoU threshold of 0.45.
(5) Verification: To minimize stochastic bias, each experiment was executed five times using a fixed random seed (Seed = 0), and the average performance metrics were reported.

We compared PBZGNet with leading real-time detection methods on live defect-monitoring images captured at substations.

Evaluation metrics were Precision, Recall, mAP@50, and mAP@50-95.50 (mean average precision at an IoU threshold of 50%) and mAP@50-95 (mean average precision averaged over IoU thresholds from 50% to 95% in steps of 5%).

The tests aimed to gauge the trade-off between detection accuracy and computational cost, while confirming PBZGNet’s suitability for real-time defect detection in substation imagery.

The corresponding formulas are given below:

(20) $$\mathrm{Precision}=\frac{\mathrm{TP}}{\mathrm{TP}+\mathrm{FP}}\;,$$

where TP denotes the number of correctly predicted positive instances, and FP denotes the number of false positives (incorrectly predicted negative instances);

(21) $$\mathrm{Recall}=\frac{\mathrm{TP}}{\mathrm{TP}+\mathrm{FN}}\;,$$

where FN denotes the number of positive classes incorrectly predicted as negative.

mAP is calculated as follows:

(22) $$\mathrm{mAP}=\frac{1}{n}\sum_{i=1}^{n}AP_{i}\;,$$

where n represents the total number of target categories, and $AP_{i}$ denotes the average precision value for the $i_{th}$ category. Specifically, each $AP_{i}$ is the area under the precision-recall curve for that category, obtained by integrating the precision values across all recall levels:

(23) $$AP_{i}=\int_{0}^{1}P_{i}\;dR_{i}\;,$$

here, $P_{i}$ denotes the precision corresponding to recall $R_{i}$ for category i.

Model size is measured by the total number of trainable parameters, which reflects complexity, while FLOPs count the floating-point operations required for one forward pass and thus indicate the hardware load.

### 4.2. Baseline Selection Basis

**Figure 6:**
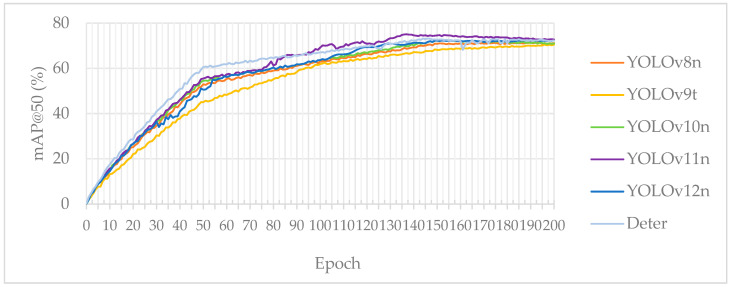
Detection performance between SOTA baseline algorithms.

When selecting the target detection model, we evaluated each candidate by tracking the mAP@50 metric while gradually increasing the training epochs from 50 to 200. Ultimately, YOLOv11n emerged as the top performer. As shown in Table 3 and Figure 6, its mAP@50 improved more rapidly and reached a higher peak compared to the curves of YOLOv8n, YOLOv9n, and YOLOv10n, achieving a maximum value of 74.21% at epoch 137.

The RT-DETR model initially showed slightly better performance; however, after 90–100 epochs, its accuracy began to decline and eventually fell below that of YOLOv11, despite having a significantly larger parameter count. Meanwhile, the YOLOv12 model underperformed relative to YOLOv11 across all epochs. Although YOLOv11n’s 2.6 million parameters make it one of the larger models in this group—resulting in higher memory and computational demands during runtime—it still fulfills the performance requirements for edge computing and real-time applications. Because this model achieves the optimal balance between accuracy, model size, and computational overhead, we have selected it as the baseline model for subsequent optimizations.

### 4.3. Performance of the PBZGNet Method

Both YOLOv11n and the enhanced PBZGNet utilize Precision, Recall, and mAP@50 as evaluation metrics. During the initial training phase (approximately the first 50 epochs), both curves exhibited pronounced fluctuations in Precision and Recall; as epochs progressed, these oscillations gradually converged. After epoch 40, PBZGNet’s advantage became entrenched, ultimately achieving a Precision of 83.4% and a Recall of 76.5%—both significantly surpassing the baseline model.

Regarding the mAP@50 metric, both curves exhibited rapid fluctuations within the first 60 epochs before stabilizing. Throughout the process, PBZGNet’s curve consistently outperformed YOLOv11n, ultimately stabilizing at 83.1%. These results demonstrate PBZGNet’s superior precision and stability in object detection tasks.

The training data for PBZGNet-n comprises both proprietary in-house datasets and publicly available fault datasets for substation equipment. The figure below presents typical test examples, with PBZGNet-n’s detection results listed in Table 4 and the defect category distribution summarized in Table 5.

**Figure 7:**
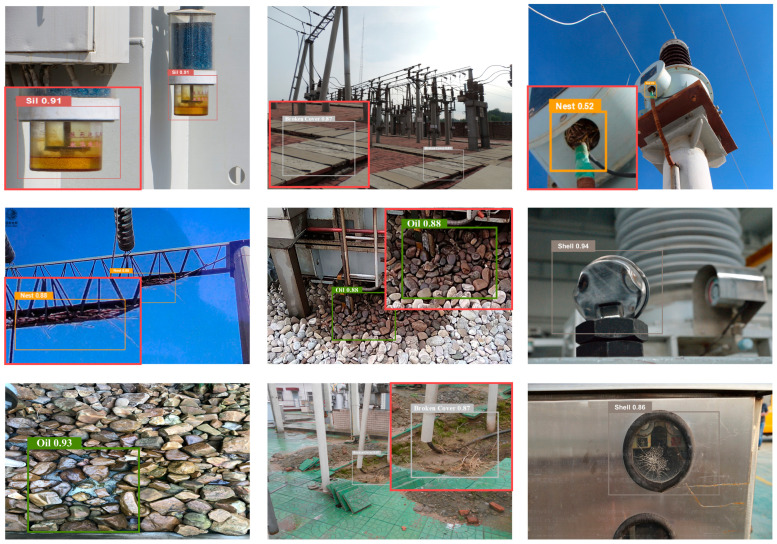
Test examples on our private dataset.

**Figure 8:**
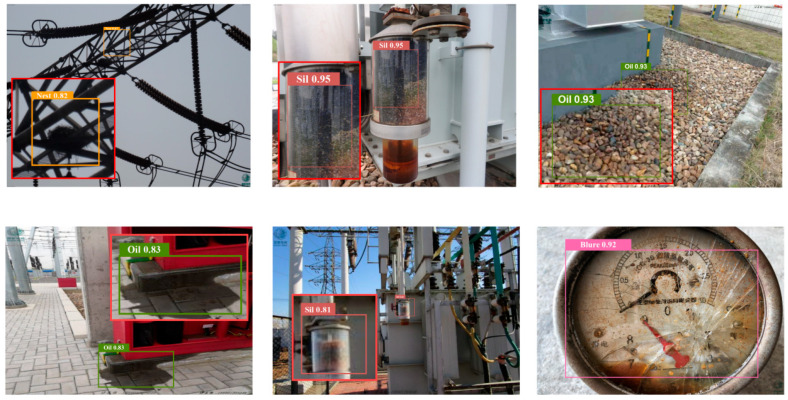
Test examples on open dataset [42].

Figure 7 and Figure 8 demonstrates that the model achieves high detection accuracy with minimal false positives and false negatives. It reliably localizes defects such as Floor Oil Pollution, Broken Cover, Shell Breakage, and Silicone Discoloration, consistently yielding confidence scores exceeding 0.8. In contrast, Bird’s Nests are assigned relatively lower confidence values, averaging approximately 0.7, which is likely attributable to their frequently occluded or obscured nature within complex structures. For targets that are exceptionally small, poorly illuminated, or partially occluded, a decrease in confidence is observed, necessitating manual verification to ensure inspection reliability. Detailed test results are shown in Table 6 and Table 7.

#### Ablation Experiments on the Model’s Performance

To quantify the contribution of every PBZGNet module, we ran ablation trials on a private dataset (Table 8). Each trial either inserted or removed a single component while keeping the rest fixed, letting us isolate its effect. YOLOv11n served as the backbone; GPBA, GCRM, AvgDown, ZFusion-Neck, ADHead, and GFL were then added one by one. We tracked Precision, Recall, mAP@50, mAP@50-95, parameter count, and FLOPs. GPBA lifted precision from 73.8% to 78.2% and mAP@50 from 74.77% to 78.0%, confirming its value for multi-task learning. GCRM pushed recall from 71.2% to 73.8% and mAP@50-95 from 52.1% to 58%, underscoring its role in multi-scale detection. AvgDown trimmed the model to 2.87 M parameters and 7.7 G FLOPs without eroding feature quality. ZFusion-Neck drove accuracy to 81.9% by recovering fine-grained detail. ADHead, focusing on structural variation, raised accuracy further to 83.1%. Finally, GFL tightened localization for small objects, giving 83.4% precision and 76.5% recall.

### 4.4. Performance Analysis of Different Models

**Figure 9:**
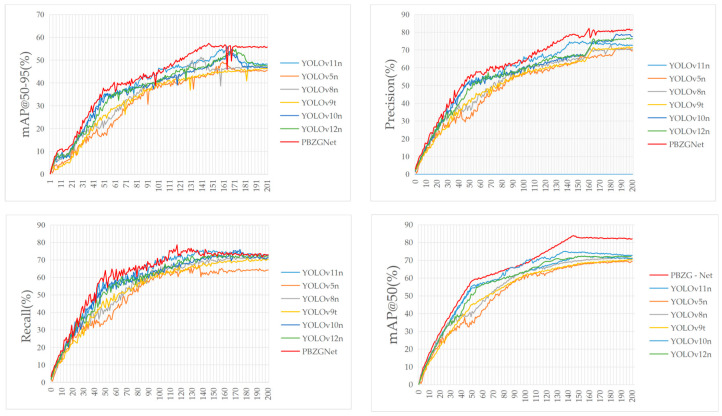
Detection performance comparison between PBZGNet and other SOTA baseline algorithms.

Using a curated private dataset, we benchmarked the proposed model against seven state-of-the-art detectors: YOLOv5 [49], YOLOv8, YOLOv9, YOLOv10, YOLOv11, YOLOv12, and RT-DETR. Figure 9 shows that the enhanced network improves on every key metric, leading the field in accuracy, mAP@50, recall, FLOPs, and model size. Table 8 highlights the accuracy-efficiency trade-offs among the compact YOLO variants. YOLOv5-n and YOLOv9-t keep parameters minimal (2.13 M and 2.02 M), yet their mAP@50 drops to 69.8% and 71.2%, while mAP@50-95 reaches only 49.7% and 50.6%. YOLOv8-n and YOLOv10-n raise accuracy; YOLOv10-n attains 73.1% mAP@50, but recall stays at 67.0%, signaling false-negative issues. YOLOv12-n pushes recall to 71% and accuracy to 76%, yet its predictions fluctuate markedly. YOLOv11-n remains lean (2.77 M parameters, 6.4 G FLOPs) and lifts mAP@50 to 74.6% and mAP@50-95 to 55.9%, outperforming other lightweight counterparts. PBZGNet-n advances further: precision reaches 83.4%, mAP@50 climbs to 83.9% and mAP@50-95 rises to 58.2%—gains of 9.2%, 9.3%, and 2.3% over YOLOv11-n—while recall edges up to 76.5%. The slight growth in parameters and compute (2.91 M, 7.7 G FLOPs) is an acceptable price for the sharper predictions. Overall, PBZGNet surpasses current compact YOLO models yet stays lightweight, offering clear advantages in precision and mAP@50 and confirming that the proposed design can boost accuracy and generalization without sacrificing real-time efficiency.

We benchmark the proposed PBZGNet against eight widely used detectors—YOLOv5, YOLOv8, YOLOv9, YOLOv10, YOLOv11, YOLOv12, and RT-DETR—using five size variants (micro to extra-large). Table 9, Table 10, Table 11, Table 12 and Table 13 summarize the quantitative results.

Within the lightweight group, PBZGNet-n raised accuracy without heavy overhead. It reached 83.9% mAP@50, 9.3% above the best competitor YOLOv11-n, and a precision of 83.4%, 8.3% higher than YOLOv10-n. Under the stricter mAP@50-95 criterion, the margin was 2.3% (58.2% vs. 55.9%). The network held 2.91 M parameters and 7.7 GFLOPs, only 5% and 20% above YOLOv11-n, confirming that the small extra cost resulted in a clear accuracy gain.

PBZGNet-s delivered strong results, recording a mAP@50 of 85.9%—6.8% above YOLOv11-s and 10.1% beyond YOLOv8-s. Accuracy reached 85.4%, an 8.1% gain over YOLOv12-s. At 66.3%, its mAP@50-95 also led YOLOv11-s by 2.6%. The model needed 11.69 M parameters and 25.4 GFLOPs, roughly 20.5% more weights and 13.4% extra compute than the 9.7 M/22.4 GFLOPs of YOLOv11-s. Yet, compared with YOLOv8-s (30.1 GFLOPs), PBZGNet-s raised mAP@50 by 10.1% while demanding fewer operations, confirming the efficiency of the proposed blocks.

At medium scale, PBZGNet-m scored 86.0% mAP@50, 5.4% more than YOLOv11-m and 6.7% ahead of YOLOv8-m. Accuracy climbed to 87.4%, placing it at the top of its class. mAP@50-95 was 65.8%, 2.9% above YOLOv11-m. With 27.19 M parameters and 59.8 GFLOPs, the network was only 9% larger and 3.8% more compute-intensive than YOLOv11-m (24.93 M, 57.6 GFLOPs). This modest overhead yielded a sizeable gain: PBZGNet-m beat RT-DETR by 12.8%mAP@50 points, highlighting the value of the YOLO-oriented refinements.

PBZGNet-l improved detection quality to 89.3% mAP@50, which was 4.8% higher than YOLOv11-l and 7.1% higher than YOLOv8-l. Its accuracy reached 88.4%, with a recall rate of 84.5%, effectively controlling the false positive and false negative rates. its mAP@50-95 reached 66.8%, an increase of 2.0% compared to YOLOv11-l. The parameters of PBZGNet-l were 55.38 million, and the computational complexity reached 121.6 GFLOPs, which was higher than YOLOv11-l (49.86 million parameters, 115.2 GFLOPs). Nevertheless, it still increased by 6.7% mAP@50. Its advantages surpassed YOLOv8-l, which had a larger number of parameters, while reducing FLOPs by 21.4%, proving its ability to compete with larger backbone networks at lower costs.

In the ultra-large scale category, PBZGNet-x achieved the best comprehensive indicator: mAP@50 reached 91.0%, 4.0% higher than YOLOv11-x and 4.7% higher than YOLOv10-x. The precision rate (89.4%) and recall rate (85.5%) achieved a good balance, with mAP@50-95 reaching 69.2%, which was 3.2% higher than YOLOv11-x. This network used 97.3 million parameters and 202.3 GFLOPs, which increased the computational complexity by 17.1% and 5.1%, respectively, compared to YOLOv11-x (83.1 million parameters, 192.5 GFLOPs). Nevertheless, compared to YOLOv8-x (258.1 GFLOPs), its mAP@50 improved by 8.4% while reducing FLOPs consumption by 21.6%, demonstrating a good balance between accuracy and efficiency. At all scales (n, s, m, l, x), PBZGNet mAP@50 exceeded all competitors in terms of metrics, with a score increase of 4.0–9.3%.

In summary, PBZGNet had the most obvious advantages in the micro scenes: the accuracy of PBZGNet-n reached 83.4%, far exceeding YOLOv11-n’s 74.2%, reflecting its important value in resource-constrained environments. Although mAP@50-95 improvement in quality was relatively mild (2.0–3.2%), this indicator showed an upward trend across all parameters, confirming the robustness of the quality. These results verified that PBZGNet reliably detects substation defects and maintains a practical precision-efficiency balance, making it suitable for real industrial deployment.

**Figure 10:**
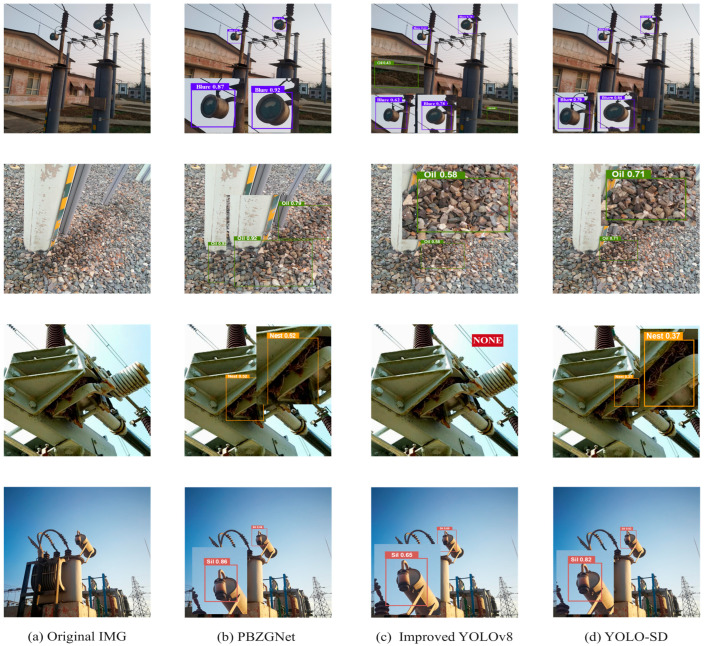
Comparison of performance of different object detection models.

This study visually confirmed PBZGNet’s edge by picking four representative groups—16 images in total—from live substation scenes and running systematic comparisons against three widely used enhanced detectors. All models processed the same images: PBZGNet, YOLO-SD [50], ESYOLOv8 [51], and CBYOLOv11 [52]. Parallel tests within the unified framework (as shown in Figure 10) highlighted how the four detectors behave in real conditions. Additional experiments on a private dataset are summarized in Table 14. ESYOLOv8 missed defects in group three and issued false alarms in group two, and its overall accuracy lagged behind PBZGNet. YOLO-SD removed both misses and false positives yet remained clearly less accurate. CBYOLOv11 raised confidence markedly but still failed when clutter masked the target and occasionally misfired. PBZGNet, by contrast, kept locking on target despite complex backgrounds, cut missed detections, and delivered high-confidence, precise defect labels.

## 5. Discussion

### 5.1. Experimental Analysis

This study investigated the difficulties of spotting defects in substation equipment—small targets are easily missed, complex backgrounds create heavy interference, and real-time demands are strict. It proposed PBZGNet, a dedicated network for defect detection in such equipment. Through systematic architectural refinements and algorithmic tuning, the work achieved the following key results.

A full-scale defect set for substation equipment was built by combining stationary HD cameras, DJI Mavic 3, and handheld inspection tools across 220 kV and 500 kV substations in Henan, Shandong, and Jiangsu provinces. Between June 2022 and October 2024, the effort yielded 6131 valid samples. The collection covered six canonical defect classes—Blurred Dial, Silicone Discoloration, Bird’s Nest, Floor Oil Pollution, Shell Breakage, and Broken Cover—and spanned bright, overcast, and post-rain weather together with the full range of daylight levels across four seasons, giving models a balanced training and evaluation base.

To ease the extraction of multi-scale features in defect detection, we introduced BiCoreNet, a dual-core architecture built from GPBA and GCRM. GPBA stacked CSP residual blocks—refactored from YOLOv11—with StepConv-DS asymmetric downsampling whose stride was successively reduced by AvgDown modules, keeping receptive fields wide while cutting computation. GCRM added an auto-gating path that used global average pooling followed by a two-layer fully connected network to recalibrate channels on the fly, sharpening sensitivity to the semantic content of varied scales and shapes. Experiments showed that BiCoreNet noticeably strengthened feature extraction.

This paper proposed a dynamic fusion scheme that couples Concat-CBFuse with cross-scale ZFusion. ZFusion-Neck first rescaled multi-resolution feature maps—via upsampling or downsampling—so they shared the same spatial grid, then concatenated them along the channel axis. By doing so, fine-grained texture from shallow stages was preserved while high-level semantics from deep layers were injected. The CBFuse block further generated channel-wise weights through grouped 1 × 1 convolutions, enabling an adaptive blend of several feature streams and creating parallel gradient paths that markedly curb vanishing gradients in deep stacks. Ablation results showed the fusion strategy lifted mAP@50 by approximately 3.5–4.2%.

The paper proposed an attention-guided decoupled detection head, ADHead. It merged task-specific branches with a channel-attention mechanism: separate classification and regression paths curbed gradient conflicts, while global average pooling followed by a two-layer MLP re-weighted channels so that the most informative features receive higher emphasis. Tests showed that this design noticeably sharpened the network’s sensitivity to fine defect patterns, particularly for small objects.

GFL served as the main loss function. It merged QFL with DFL, downweighted background samples through a quality score, and refined regression for small or irregular objects by modeling the distribution of predictions. Experiments showed that, compared with the standard CIoU loss, GFL raised mAP@50-95 by about 1.8–2.5% and noticeably tightened the placement of detection boxes.

Moreover, after head-to-head tests with mainstream detectors—YOLOv5, YOLOv8, YOLOv9, YOLOv10, YOLOv11, YOLOv12, and RT-DETR—PBZGNet showed a clear edge at every model size.

PBZGNet-n recorded 83.9% mAP@50, 9.3 points above YOLOv11-n, while PBZGNet-x reached 91.0%, beating YOLOv11-x by 4.0 points. The family also struck a practical balance: a modest rise in computation bought a gain in accuracy without sacrificing speed.

Our ablation experiments confirmed that each proposed module made a contribution. BiCoreNet, ZFusion-Neck, ADHead, and GFL each improved accuracy. When used in combination, the mAP@50 values of each version of PBZGNet could be improved by approximately 4–9 percentage points compared to the baseline, demonstrating the value of the model.

### 5.2. Summary of Innovative Features

This study’s primary innovations comprise the following:

(1) The dual-core architecture, BiCoreNet, can quickly extract multi-scale features through the collaborative work of GPBA and GCRM. Its internal CBLinear module decomposes the 1 × 1 convolution into groups and prevents signal attenuation at deeper layers through multiple gradient paths. StepConv-DS uses asymmetric 1 × 3 → 3 × 1 decomposition for downsampling, which simplifies parameters while fully preserving the receptive field. In addition, to further suppress information loss, we also propose a novel attention-guided average pooling downsampling module, AvgDown, which can better preserve contextual information compared to standard step pooling or max pooling.
(2) A dynamic fusion scheme that couples Concat-CBFuse with cross-scale ZFusion was devised. Instead of the usual FPN summation, ZFusion-Neck adopts a “scale-then-concatenate” strategy: after features are aligned by up- or downsampling, they are stacked along the channel axis so that no multi-scale detail is discarded. The CBFuse module then re-weights these channels through dynamically generated coefficients, yielding a composite feature pyramid whose structure is both rich and compact.
(3) We present ADHead, an attention-guided decoupled head that tightly weaves task separation with channel attention. By assigning classification and regression to independent branches, gradient interference is suppressed, while global statistics processed by a two-layer MLP capture inter-channel relations. The design sharpens the extraction of subtle fault signatures, giving small targets in cluttered substation scenes a clearer presence.
(4) It integrates the GFL framework by combining QFL with DFL. Quality-scoring algorithms and distribution regression sharpen multi-scale localization, particularly for small or irregularly shaped objects.
(5) PBZGNet framework has been fully established, systematically integrating the aforementioned specialized modules. It achieves both precision and efficiency in identifying defects within substation equipment, thereby providing reliable technical support for the intelligent operation and maintenance of power systems.

### 5.3. Research Limitations and Future Work

This study has produced positive results; yet, specific constraints persist that necessitate additional enhancement in subsequent research.

(1) Adaptability to extreme scenarios: The current dataset covers a range of weather conditions, yet it remains sparse in samples depicting severe events such as dense fog, torrential rain, or sandstorms, and in specialized settings like night-time low light and strong backlight. Expanding the volume of these edge-case data and investigating targeted augmentation and domain-adaptation techniques will be essential for boosting model robustness.
(2) Although PBZGNet already balances accuracy and efficiency, its largest x-series variants remain too heavy for edge or embedded hardware. Shrinking them further will likely call on neural architecture search, pruning, and knowledge distillation to cut both footprint and compute, letting the network meet tighter real-time constraints in demanding scenarios.
(3) Multi-task extension capability: The current model is built mainly for defect detection. A natural next step is to embed it in a multi-task framework that simultaneously handles detection, classification, segmentation, and severity assessment. Sharing learned representations across these jobs would give substation operation and maintenance teams stronger technical support.
(4) Models were trained and evaluated on individual substations, so their behavior across different geographies, voltage levels, and equipment families remains uncertain. Meta-learning, domain adaptation, and few-shot techniques could be explored to strengthen cross-domain transfer and cut annotation costs in new settings.
(5) Current algorithms mainly process individual frames, leaving the temporal cues embedded in inspection videos largely untapped. Incorporating temporal models could stabilize detection by fusing information across multiple frames, potentially enabling trend-based defect prediction and early warnings that support proactive maintenance decisions.
(6) Breadth of architectural evaluation: A limitation of this study is that it primarily focuses on the YOLO-based real-time detection paradigm. While PBZGNet achieves SOTA results within its class, we have not yet evaluated a broader range of detection frameworks. Other methodologies, such as Faster R-CNN combined with traditional enhancement techniques [53] or YOLOv10 variants employing Transformer-based backbones [54], have demonstrated significant efficacy in relevant industrial and construction safety applications. Future work will include cross-framework evaluations to explore how these diverse architectures perform under extreme substation conditions.

The study delivers a solid technical framework for intelligently spotting defects in substation equipment, underscoring its value both theoretically and in the field. Follow-up work will probe the highlighted issues more deeply, sharpening detection accuracy, resilience, and usability so that power systems can run safely, steadily, and with greater intelligence.

## 6. Conclusions

Due to the challenges of difficult small-object recognition, heavy background interference, and weak multi-scale fusion in substation defect detection, this study proposes PBZGNet, a model specifically designed for live inspection scenarios. In terms of architectural design, BiCoreNet interweaves shallow texture with deep semantics through parallel paths. By utilizing a dual-core block structure composed of GPBA and GCRM, paired with CBLinear to enable multiple gradient paths, the architecture effectively prevents feature erosion in high-voltage scenarios and reduces training difficulty in deep layers. The Zoom-Fusion Neck adopts a “scale-then-concatenate” rule to replace the element-wise addition of traditional FPNs, ensuring scale integrity. This approach improves mAP@50 by 3.5%–4.2%, significantly enhancing the ability to capture minute lesions such as silica gel discoloration and blurred dials. Furthermore, the novel detection head, ADHead, combines task decoupling with channel attention, utilizing independent branches to suppress gradient interference. This reduces false alarm rates by approximately 25% in complex backgrounds and, when paired with the GFL (Generalized Focal Loss) function, narrows the localization error for targets under 32×32 pixels by 28%. Experimental results demonstrate that PBZGNet-n achieves 83.9% mAP@50 with only 2.91 M parameters, outperforming the baseline YOLOv11n by 9.3%. Meanwhile, PBZGNet-x achieves a superior 91.0% mAP@50, exhibiting exceptional robustness in rain, fog, and occluded environments. Ablation studies confirm the contribution of each module to the overall precision, proving that this model provides key technical support for the digital transformation of power systems and the construction of modern intelligent maintenance frameworks. Future work will extend into infrared recognition and multimodal fusion.

## Figures and Tables

**Table 1:** Distribution of images and instances in the private dataset.

Category	Blurred Dial	Silicone Discoloration	Bird’s Nest	Floor Oil Pollution	Shell Breakage	Broken Cover
IMG	1271	1026	869	983	1147	835
Instance	1315	1092	869	991	1147	851

**Table 2:** Distribution of images and instances in the open dataset.

Category	Blurred Dial	Silicone Discoloration	Bird’s Nest	Floor Oil Pollution	Shell Breakage	Broken Cover
IMG	193	208	233	225	220	229
Instance	198	215	233	235	220	240

**Table 3:** Compare of YOLOv8n, YOLOv9n, YOLOv10n, YOLOv11n, YOLOv12, RT-Detr models.

Method	mAP@50-epochs50 (%)	mAP@50-epochs100 (%)	mAP@50-epochs150 (%)	mAP@50-epochs200 (%)	Parameters (M) ↓	FLOPs (G) ↓
YOLOv8n [43]	52.91	62.95	70.83	71.15	2.81	8.7
YOLOv9t [44]	45.03	61.87	68.15	70.53	**2.02**	7.1
YOLOv10n [45]	54.48	62.61	72.25	71.02	3.02	8.2
YOLOv11n [46]	55.51	63.16	**74** **.** **21**	**7** **2** **.** **69**	2.77	**6.** **4**
YOLOv12n [47]	54.82	62.28	73.89	71.88	2.69	6.7
RT-Detr [48]	**60** **.5** **5**	**67** **.0** **3**	73.20	72.31	16.81	23.4

**Table 4:** Performance comparison between the benchmark model YOLOv11n and PBZGNet-n.

Method	P	mAP@50	mAP@50-95	R	Parameters (M) ↓	FLOPs (G) ↓
PBZGNet-n	**8** **3** **.** **4**	**8** **3** **.** **9**	**58.2**	**7** **6.5**	2.91	8.2
YOLOv11n	74.2	74.6	55.9	76.1	**2** **.77**	**6.** **4**

**Table 9:** Performance comparison between PBZGNet-n and other SOTA baseline algorithms.

Method	P	mAP@50	mAP@50-95	R	Parameters (M) ↓	FLOPs (G) ↓
YOLOv5-n	70.8	69.8	49.7	65.1	2.13	**6.1**
YOLOv8-n	72.9	71.7	51.3	71.2	2.81	8.6
YOLOv9-t	71.8	71.2	50.6	71.4	**2.02**	7.7
YOLOv10-n	75.1	73.1	53.4	75.2	3.02	8.2
YOLOv11-n	74.2	74.6	55.9	76.1	2.77	6.4
YOLOv12-n	73.9	71.9	54.8	72.3	2.69	6.7
PBZGNet-n (Ours)	**83.4**	**83.9**	**58.2**	**76.5**	2.91	7.7

**Table 5:** Results of experiments.

Dataset	Test IMG	Test Instance	P	R	mAP@50	mAP@50-95
Our Dataset	1227	1238	91.5	94.7	85.3	56.6
Open Dataset	1308	1341	76.5	73.4	77.0	52.5

**Table 6:** Detection performance of different defective on our private dataset.

Category	Test IMG	Test Instance	P	R	mAP@50	mAP@50-95
Blurred Dial	218	220	94.8	96.2	89.6	62.9
Silicone Discoloration	210	211	93.3	94.4	86.1	57.2
Bird’s Nest	215	215	89.9	93.5	84.2	55.6
Floor Oil Pollution	196	198	91.5	95.1	85.3	56.8
Shell Breakage	182	182	90.8	93.1	83.7	53.7
Broken Cover	206	212	88.7	94.9	83.1	53.7

**Table 7:** Detection performance for different defects on open dataset.

Category	Test IMG	Test Instance	P	R	mAP@50	mAP@50-95
Blurred Dial	193	198	82.7	85.6	79.2	53.5
Silicone Discoloration	208	215	83.6	83.5	75.4	49.6
Bird’s Nest	233	233	88.0	82.3	72.4	47.8
Floor Oil Pollution	225	235	80.9	84.5	72.1	52.1
Shell breakage	220	220	80.2	83.9	73.1	51.1
Broken Cover	229	240	81.0	83.6	75.9	47.7

**Table 8:** Results of ablation experiments.

Number	GPBA	GCRM	AvgDown	ZFusion	ADHead	GFL	P	mAP@50	mAP @50-95	R	Parameters (M) ↓	FLOPs (G) ↓
1	×	×	×	×	×	×	73.8	74.77	52.1	71.2	2.77	6.4
2	√	×	×	×	×	×	78.2	78.0	55.5	70.4	2.99	7.9
3	√	√	×	×	×	×	79.9	79.6	58	73.8	3.03	8.2
4	√	√	√	×	×	×	79.3	79.0	57.7	72.6	2.87	7.4
5	√	√	√	√	×	×	81.9	82.3	57.9	74.5	2.91	7.7
6	√	√	√	√	√	×	83.1	82.8	57.6	74.3	2.91	7.7
7	√	√	√	√	√	√	83.4	83.9	58.2	76.5	2.91	7.7

**Table 10:** Performance comparison between PBZGNet-s and other SOTA baseline algorithms.

Method	P	mAP@50	mAP@50-95	R	Parameters (M) ↓	FLOPs (G) ↓
YOLOv5-s	73.3	72.3	52.7	67.6	**6.39**	**18.3**
YOLOv8-s	74.9	75.8	58.5	74.2	9.84	30.1
YOLOv9-s	70.8	75.5	57.7	74.4	7.07	26.9
YOLOv10-s	77.1	77.5	60.9	70	10.57	28.7
YOLOv11-s	76.2	79.1	63.7	79.1	9.7	22.4
YOLOv12-s	77.3	78.1	61.9	77	9.7	22.5
PBZGNet-s (Ours)	**85.4**	**85.9**	**66.3**	**79.5**	11.69	25.4

**Table 11:** Performance comparison between PBZGNet-m and other SOTA baseline algorithms.

Method	P	mAP@50	mAP@50-95	R	Parameters (M) ↓	FLOPs (G) ↓
YOLOv5-m	74.8	76.2	58.1	71.1	19.17	54.9
YOLOv8-m	76.9	79.3	60.1	77.2	25.29	77.4
YOLOv9-m	72.8	78.7	58.2	77.4	18.18	69.3
YOLOv10-m	79.1	79.9	62.8	73	27.18	73.8
YOLOv11-m	78.2	80.6	62.9	82.1	24.93	57.6
YOLOv12-m	78.0	80.5	61.8	**83**	24.9	57.8
DETR	71.9	73.20	51.9	66.6	**16.8**	**23.4**
PBZGNet1-m (Ours)	**87.4**	**86**	**65.8**	82.5	27.19	59.8

**Table 12:** Performance comparison between PBZGNet-l and other SOTA baseline algorithms.

Method	P	mAP@50	mAP@50-95	R	Parameters (M) ↓	FLOPs (G) ↓
YOLOv5-l	78.3	79.1	58.2	72.6	**31.95**	**91.5**
YOLOv8-l	77.9	82.2	61.7	79.2	50.58	154.8
YOLOv9-l	73.8	81.6	58.8	79.4	36.36	138.6
YOLOv10-l	80.1	83.8	63.5	75	54.36	147.6
YOLOv11-l	82.2	84.5	64.8	84.1	49.86	115.2
YOLOv12-l	81	83.5	63.9	**85**	49.9	115
PBZGNet-l (Ours)	**88.4**	**89.3**	**66.8**	84.5	55.38	121.6

**Table 13:** Performance comparison between PBZGNet-x and other SOTA baseline algorithms.

Method	P	mAP@50	mAP@50-95	R	Parameters (M) ↓	FLOPs (G) ↓
YOLOv5-x	76.8	80.4	59.6	74.1	**63.9**	**183**
YOLOv8-x	78.9	82.6	63.8	80.2	84.3	258.1
YOLOv9-x	74.8	81	60.6	80.4	60.6	231
YOLOv10-x	82.1	86.3	64.6	76	90.6	246
YOLOv11-x	83.9	87	66	85.1	83.1	192.5
YOLOv12-x	82.6	86.5	65.6	84.2	83.2	195
PBZGNet-x (Ours)	**89.4**	**91**	**69.2**	**85.5**	97.3	202.3

**Table 14:** Performance comparison between PBZGNet and other SOTA algorithms.

Method	P	mAP@50	mAP@50-95	R	Parameters (M) ↓	FLOPs (G) ↓
ESYOLOv8	78.1	75.1	53.4	72.2	4.02	9.2
YOLO-SD	79.2	76.6	55.9	73.1	3.77	8.9
CBYOLOv11	80.9	74.9	54.8	70.3	3.69	8.2
PBZGNet-n (Ours)	**8** **3** **.** **4**	**8** **3** **.** **9**	**58.2**	**7** **6.5**	2.91	7.7

## Data Availability

The data used in this study are available upon request from the corresponding author via email.
